# The association between acute flaccid myelitis (AFM) and Enterovirus D68 (EV-D68) – what is the evidence for causation?

**DOI:** 10.2807/1560-7917.ES.2018.23.3.17-00310

**Published:** 2018-01-18

**Authors:** Amalie Dyda, Sacha Stelzer-Braid, Dillon Adam, Abrar A Chughtai, C Raina MacIntyre

**Affiliations:** 1 School of Public Health and Community Medicine, University of New South Wales (UNSW), Sydney, New South Wales (NSW), Australia; 2 School of Medical Sciences, University of New South Wales (UNSW), Sydney, New South Wales (NSW), Australia; 3 Division of Serology and Virology, South Eastern Area Laboratory Services, Prince of Wales Hospital, Sydney, Australia; 4 College of Public Service and Community Solutions and College of Health Solutions, Arizona State University, Tempe, Arizona, United States

**Keywords:** Enterovirus D68, acute flaccid myelitis, acute flaccid paralysis, Bradford Hill Criteria, AFM, EV-D68

## Abstract

Enterovirus D68 (EV-D68) has historically been a sporadic disease, causing occasional small outbreaks of generally mild infection. In recent years, there has been evidence of an increase in EV-D68 infections globally. Large outbreaks of EV-D68, with thousands of cases, occurred in the United States, Canada and Europe in 2014. The outbreaks were associated temporally and geographically with an increase in clusters of acute flaccid myelitis (AFM). **Aims:** We aimed to evaluate a causal association between EV-D68 and AFM.  **Methods:** Using data from the published and grey literature, we applied the Bradford Hill criteria, a set of nine principles applied to examine causality, to evaluate the relationship between EV-D68 and AFM. Based on available evidence, we defined the Bradford Hill Criteria as being not met, or met minimally, partially or fully.  **Results:** Available evidence applied to EV-D68 and AFM showed that six of the Bradford Hill criteria were fully met and two were partially met. The criterion of biological gradient was minimally met. The incidence of EV-D68 infections is increasing world-wide. Phylogenetic epidemiology showed diversification from the original Fermon and Rhyne strains since the year 2000, with evolution of a genetically distinct outbreak strain, clade B1. Clade B1, but not older strains, is associated with AFM and is neuropathic in animal models.  **Conclusion:** While more research is needed on dose–response relationship, application of the Bradford Hill criteria supported a causal relationship between EV-D68 and AFM.

## Background

Large outbreaks of enterovirus D68 (EV-D68), affecting at least 2,287 people, occurred in multiple countries in 2014. The first outbreak was detected in the United States (US), followed by Canada, Europe and Asia. At the same time, an increase in clusters of acute flaccid myelitis (AFM) occurred in the same geographical areas, with the highest number of cases (n = 120) in the US [1-3]. Smaller numbers of AFM cases (at least six) associated with EV-D68 infection were reported from Denmark, France, the Netherlands, Spain, Sweden and the United Kingdom (UK) [1-3]. This raised the question of causal association between EV-D68 and AFM [1-3]. 

The enterovirus (EV) genus comprises 12 distinct species [4], including poliovirus. EV-D68 belongs, with other non-polio EVs, to the group EV D [5,6]. AFM is defined as ‘acute flaccid paralysis in one or more limbs or acute onset of bulbar paralysis’ and if caused by poliovirus, is referred to as poliomyelitis or polio [7]. In addition to poliovirus as the leading cause of AFM [8,9], other EVs such as EV-A71 are also a recognised cause of AFM [10,11]. Following the 2014 epidemic, EV-D68 has emerged as another possible cause of AFM [12].

Infection with EV-D68 historically caused mild respiratory symptoms such as rhinorrhoea, muscle aches and cough. Recently however, it has been associated with severe respiratory symptoms, hospitalisation and death [13]. Children are at higher risk of symptomatic infection than adults [2]. Transmission occurs from person to person, and the virus is found in respiratory secretions, blood and infrequently in cerebrospinal fluid (CSF) [13,14].

EV-D68 was first identified in 1962 and has since been found sporadically and in small clusters, with only 699 confirmed cases worldwide until 2013 [15,16]. From 2008 to 2010, the US Centers for Disease Control and Prevention (CDC) reported the identification of six clusters of respiratory illness associated with EV-D68 in Asia, Europe and the US, with the number of confirmed cases ranging from five to 28 [2]. From 2009 to 2013, a total of 79 cases were reported in the US, with generally mild symptoms [1], followed by 1,153 cases in 2014 [13]. In addition, an outbreak involving 25 people occurred in June 2016 in the Netherlands [17].

The global prevalence of EV-D68 as a cause of illness appears to be low. Studies have reported the proportion of respiratory specimens positive for EV-D68 ranging from 0.2 to 3.4% [18-21]. A study conducted from 2011 to 2015 in China found that 12 of 7,945 (0.2%) specimens were positive for EV-D68 [21]. In Hong Kong an investigation in children showed that 24 of 1,461 (1.6%) of respiratory samples were positive for EV-D68 [19]. In Germany, where respiratory samples were collected from patients in three hospitals between January 2013 and December 2014 [18], 39 of 14,838 (0.3%) were positive for EV-D68 [18]. Data from the French enterovirus surveillance network showed that from July to December 2014, 212 of 6,229 (3.4%) samples were positive for EV-D68 [20]. However, prevalence may be affected by geographical location, type of test conducted and testing practices (such as changes in likelihood of testing for EV-D68 during an epidemic, owing to increased awareness among clinicians of the disease and the availability of tests).

The first detected large-scale outbreak of EV-D68 was reported in the US and Canada in 2014 [3,22,23] and was associated with severe respiratory symptoms [13]. The outbreak occurred from August 2014 to January 2015 (autumn/winter), with a total of 1,153 cases in 49 US states [13]. The disease severity was much higher than in previous outbreaks, with higher numbers of people experiencing severe respiratory disease. AFM was diagnosed in 10.4% (120/1,153) of cases in the US and five cases of neurological illness were associated with EV-D68 in Canada [11,22,24,25].

Outbreaks were also reported in Europe. Investigation into an increase in the number of children with severe respiratory disease in September 2014 in a hospital in Norway found that 33 of 303 (10.9%) paediatric samples were positive for EV-D68 [26]. The hospital reported two cases of severe AFM associated with EV-D68 infection [27]. A sporadic case of AFM following EV-D68 infection was also reported in France [28] and the UK in 2014 [29].

Phylogenetic analysis of a section of the EV-D68 genome (the VP1 gene) has identified four distinct clades of EV-D68, named A, B, C and D [15,30]. Clade B can be further divided into B1, B2 and B3 [31]. Strains isolated in the US from the 2014 outbreak belonged mostly to clade B [30], in particular B1 [12].

Despite the growing concern following the 2014 outbreak and the association with AFM in several countries, information regarding causation between EV-D68 and AFM is still limited. We aimed to evaluate evidence of a causal relationship between EV-D68 and AFM using the Bradford Hill criteria.

## Methods

A review of the literature was conducted in April 2017 using MEDLINE and EMBASE. Systematic search terms used were ‘enterovirus 68’ OR ‘EV-D68’ or ‘EV68’ AND ‘acute flaccid myelitis’ OR ‘acute flaccid paralysis’. The search was restricted from 1980 to present. A search of the grey literature using Google was also conducted using the same search terms. All bibliographies of included studies were reviewed. Studies were included if they investigated an association between AFM and EVs, described a case series of EV-D68 and AFM or investigated the biology of EV-D68. Studies were excluded if they investigated EVs in general, i.e. not specifically EV-D68 or an association with AFM. The Bradford Hill criteria were applied to the evidence.

### Bradford Hill criteria

The Bradford Hill criteria are a set of nine criteria applied to examine causality between an exposure and a disease (Table 1) [32].

**Table 1:** Bradford Hill criteria of causality

Criterion	Description
Strength	Whether those with the exposure are at a higher risk of developing disease and if so, how much more risk? This criterion suggests that a larger association increases the likelihood of causality.
Consistency	The credibility of findings increases with repetition of findings, including consistency of study findings across different populations and geographical locations.
Specificity	Causality is more likely if the exposure causes only one specific disease or syndrome, or if a specific location or population are being affected.
Temporality	This criterion requires that the exposure must occur before the disease, and not after a latency period that is too long. This criterion must always be fulfilled for causality to be concluded.
Biological gradient	The argument for causality is stronger in the presence of a dose–response relationship, where higher or longer exposure leads to an increased risk of disease.
Plausibility	A conceivable mechanism for causation between disease and exposure should exist for there to be a causal relationship.
Coherence	The current association should not contradict any previous knowledge available about the disease and/or exposure.
Experiment	This criterion can involve scientific experiments and addresses the association of exposure with disease. However, ‘experiment’ relates to the decrease in disease risk when the exposure is removed and often involves animal models.
Analogy	This criterion uses previous evidence of an association between a similar exposure and disease outcome to strengthen the current argument for causation.

Source: [32].

### Application of the criteria for EV-D68 and AFM

The literature was reviewed to gather evidence relevant to each of the Bradford Hill criteria. Key findings from each identified study were assessed against the relevant criteria. Each criterion was qualitatively assigned one of four categories based on the amount of information available, sample size in the studies and the certainty of the findings:

The fit of the evidence to the criteria was scored as follows (Table 2):

**Table 2:** Qualitative evaluation of the Bradford Hill criteria

Fulfilment of each Bradford Hill criterion	Qualitative score
The criterion is fully met	+ + +
The criterion is partially met	+ +
The criterion is minimally met, with some aspects being consistent	+
The criterion is not met	−

Given the possible subjectivity in assigning scores, we present a summary of available evidence and justification to support the score for each criterion.

## Results

The results from the literature review are described below in the Figure.

**Figure:**
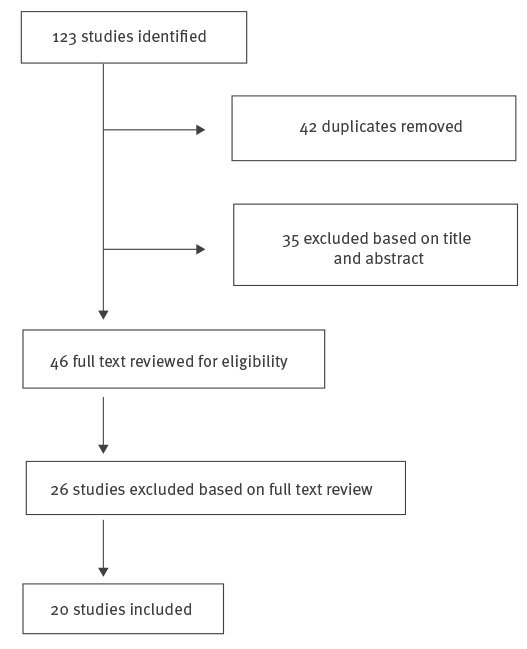
Study selection, literature review on enterovirus D68 and acute flaccid paralysis (n = 123)

The evaluation of the available evidence against the Bradford Hill criteria is summarised in Table 3 and described in more detail below to justify the final scoring.

**Table 3:** Rating of the evidence for causation between EV-D68 infection and acute flaccid myelitis

Criterion	Qualitative score
Strength	+ +
Consistency	+ + +
Specificity	+ +
Temporality	+ + +
Biological gradient	+
Plausibility	+ + +
Coherence	+ + +
Experiment	+ + +
Analogy	+ + +

### Strength

As at April 2017, there have been two epidemiological studies investigating the causation between EV-D68 infection and cases of AFM. Both were retrospective case–control studies following the 2014 outbreak in the US. The first was conducted in Colorado from 3 August to 18 October 2014. The study compared AFM cases with two control groups. Eleven cases of AFM were identified, of whom four tested positive for EV-D68. Multivariate analysis showed that those with AFM had higher odds of EV-D68 infection than both control groups (adjusted odds ratio (OR) = 10.3; 95% confidence interval (CI): 1.8–64.8 and OR = 4.5; 95% CI: 1.0–21.2) [33]. The second study was conducted in Colorado and California in patients with AFM between January 2012 and October 2014 as well as cases identified from a state-wide surveillance system in the same period [12]. EV-D68 was detected in nasopharyngeal or oropharyngeal samples in 12 of 25 patients with AFM. Among the 11 cases with AFM from two linked clusters during the 2014 outbreak, four tested positive for EV-D68. None of the total 25 AFM patients tested positive for the virus in cerebrospinal fluid (CSF). However, the association was strengthened as no other infections were identified in CSF to explain the AFM. The findings from this study are limited by the small sample size. 

A summary of these two studies is shown in Table 4 [12,33]. Given that only two studies calculated odds ratios for the association, even though convincing, we judged, based on the small number of available studies, that the criterion of strength was partially met.

**Table 4:** Summary of epidemiological studies investigating strength or consistency of association

Criterion	Study	Study period	Number of AFM cases	Proportion with EV-D68
Strength	Aliabadi et al. [33]	3 August–18 October 2014	11	4/11
	Greninger et al. [12]	2012–2014	25	12/25
Consistency	Ayscue et al. [38]	2012–2014	23	2/19
	Pastula et al. [35]	2014	9	4/9
	Messacar et al. [36]	2014	12	5/11
	Sejvar et al. [25]	2014	120	11/56
	Van Haren et al. [37]	2012–2015	59	9/45

AFM: acute flaccid myelitis; EV-D68: enterovirus D68.

### Consistency

The association between EV-D68 and AFM is generally consistent, with reports across at least nine countries (Canada, Denmark, France, the Netherlands, Norway, Spain, Sweden, the UK and the US) and different outbreaks. An increase in cases of both EV-D68 and AFM was observed in the 2014 outbreak across the US [11] and Canada [12]. A total of 120 cases of AFM were reported during this time [24,25,34]. A cluster of nine acute neurological illness cases was initially identified in Colorado in September 2014, of whom four were found to be positive for EV-D68 [35]. A second study looked at this cluster and identified 12 children with AFM, of whom 11 were tested for EV-D68; five of those 11 were positive for EV-D68 [36]. However, the strength of the above associations cannot be quantified without control data. Reports from CDC in November 2014 identified 88 cases of AFM; among the 88 cases of AFM, 41 upper respiratory tract specimens were tested, eight of which were positive for EV-D68. Of the 19 samples taken less than 14 days following symptom onset, seven tested positive for EV-D68 [37]. US national surveillance following the 2014 outbreak found 120 cases of AFM, with one CSF specimen positive and 11 of 56 upper respiratory specimens positive for EV-D68 [25]. A summary of these outbreaks is described in Table 4.

In addition, between 2012 and 2014, 23 cases of AFP were identified in California of whom two tested positive for EV-D68 [38]. Other cases of AFM have been identified in people infected with EV-D68: two in Norway [27], one in France [28] and one in the UK [29]. A study in Western Australia investigating the epidemiology of EV-D68 found three cases who developed AFM [39]. In addition, one case of AFM was identified in a cluster of 25 EV-D68 cases in the Netherlands between June and July 2016 [17].

There have also been a number of case series describing AFM and EV-D68. In a retrospective US-wide study investigating patients diagnosed with AFM during the 2014 outbreak, 11 of 56 respiratory specimens were EV-D68-positive. One of 55 CSF specimens was positive for EV-D68, however all stool/rectal swabs (n = 54) were negative. Eight of 17 cases tested within less than 7 days of symptom onset were positive for EV-D68. The low proportion of positive specimens could also be related to a delay of days between symptom onset and sampling [25]. A case series investigating AFM in California (June 2012 and July 2015) found 9 of 45 AFM cases cases infected with EV-D68 [37]. A second case series in California, from 2012 and 2014, identified 23 cases of AFP of whom two tested positive for EV-D68 [38]. A cluster of three cases of AFM were identified in 2014 in Alberta, Canada, of whom two tested positive for EV-D68 [40]. However, while these descriptive data are suggestive of a causal association, they are limited by being a small case series.

The available evidence from multiple studies showed consistency and suggested that this criterion was met.

### Specificity

Specificity in The Bradford Hill criteria can refer either to one specific exposure causing one disease or more broadly to the argument for causation being strengthened if a disease is affecting one specific population group with a similar exposure. AFM can be caused by a range of exposures and is therefore not specific to EV-D68. However, the simultaneous occurrence of EV-D68 and AFM does appear to affect a specific population, namely children: the majority of participants in a retrospective cohort study of patients of any age in the US were children diagnosed with AFM [12], while a cluster of nine cases identified in Colorado in September 2014 consisted only of people aged less than 18 years [35]. In a cluster of three cases reported in Alberta, Canada, all cases were in children aged from 5 to 15 years who had underlying respiratory conditions [40]. Other reports suggest an association between AFM and non-polio EVs which specifically affects immunocompetent children in North America [35,38].

The available evidence suggested that the criterion of specificity was partially met.

### Temporality

There is a strong temporal relationship between EV-D68 and AFM. In the 2014 US outbreak the number of EV-D68 cases increased at the same time as polio-like illness in children. In addition, the outbreak of EV-D68 subsided around the same time as the cases of AFM, with cases of both decreasing in October 2014 [25]. As mentioned previously, Sejvar et al. detected EV-D68 in eight of 17 respiratory specimens from patients with AFM collected no more than 7 days from onset of limb weakness [25].

The available evidence suggested a clear temporal relationship, with AFM following outbreaks of EV-D68 in time; the criterion of temporality was met.

### Biological gradient

There is some evidence for biological gradient. This was shown in suckling mice which were inoculated with four different strains of EV-D68 and then observed for a 2-week period [41]. The muscle and brain tissues of the mice were harvested and passaged to new mice. Limb tremors and weakness were observed after the Rhyne strain of the virus was passaged twice. Once the virus was passaged three times, the mice infected with the passaged strain developed paralysis and died [41]. 

There has been some investigation into animal models for EV-A71 infection using non-human primates: neurological symptoms in cynomolgus monkeys with EV-A71 infection were similar to those seen in humans [42]. Infection of EV-A71 in mouse models proved ineffective, owing to an incompatible murine scavenger receptor class B2 (SCARB2) receptor protein used for virus binding in humans. To circumvent this issue, Victorio et al. [43] developed a mouse-adapted EV-A71 strain. This strain induced clinical signs including paralysis and acute encephalomyelitis in 1-week-old BALB/c mice. Although this has helped increase the understanding of EV-A71 infection, an ideal animal model that can be infected with clinical EV-A71 strain has not yet been identified [44].

This criterion could be investigated further in animal models, using information on viral load where available, comparing EV-D68 viral load in patients with AFM to viral load in patients with mild EV-D68 infection. A study in 2016 concluded that, similar to rhinovirus infections, higher EV-D68 viral load may indicate more symptomatic disease [17]. Studies on viral load of EV other than EV-D68 in CSF detected an average of 10,000 to 100,000 copies/mL in adults and children [45,46]. Higher viral load was associated with increased vertigo and paraesthesia and increased leukocytes and proteins in the blood but not with fever and headache [46]. Certain EV genotypes including E30 and Coxsackie virus B (CVB 4 and 5) were also associated with increased viral loads [46]. A mouse model showed that multiple clades of EV-D68 were neurovirulent and caused paralysis, but the study did not specifically examine the dose–response relationship [47].

The available evidence from studies of other EVs and some data on EV-D68 was suggestive of a biological gradient, but because studies designed to address specifically a dose–response relationship were lacking the criterion of biological gradient was minimally met.

### Plausibility

It is biologically plausible that EV-D68 can lead to AFM. EVs are associated with neurological complications, most notably EV-A71, which has been linked to severe neurological complications including AFM and encephalomyelitis during hand foot and mouth disease (HFMD) outbreaks [48]. While EV-A71 has been shown to utilise many different receptors for facilitated cell entry, the scavenger receptor class B2 (SCARB2) protein has been most widely implicated in facilitating EV-A71 neuropathies [49]. SCARB2 is widely expressed throughout the human body, including on neurons of the CNS [50]. Furthermore, EV-A71 inoculation in a SCARB2-transgenic mouse induced encephalomyelitis clinically analogous to that observed in humans [51]. In contrast, CV-A16, which can similarly bind to SCARB2 for entry into cells, is not a neurotropic virus [49]. This suggests either that EV-A71 uses an alternative receptor for neurovirulence or that other unknown factors may inhibit entry of CV-A16, but not EV-A71, into neurons [50].

While it is known that D68 binds to cells in the respiratory tract via sialic acid receptors, the molecular basis its neurotropic pathway has yet to be established [52,53]. Like for EV-A71, the neurotropic pathway and subsequent neuropathies of EV-D68 may be an infrequent diversion from the normal replication cycle in the respiratory mucosa. There is evidence that EV-D68 is capable of infecting nerve cells, with one study showing virus detected in the CSF of a young adult with AFM in 2005 [14]. In 2008, EV-D68 was detected during autopsy in the brain and CSF of a 5-year-old boy with fulminant encephalitis [54]. In a 2015 study, the virus was detected in CSF from a 25 year-old man [39]. A retrospective study of the 2014 outbreak in the US also found a CSF specimen positive for EV-D68 in a patient with AFM [25]. However, despite these examples, most cases of AFM associated with EV-D68 have been negative for the virus in CSF. Both poliovirus and EV-A71 are rarely detected in the CSF, even among cases with AFM [55,56]. Poliomyelitis is typically confirmed by detection of virus in the stool, while EV-A71 is usually detected in the respiratory tract [25]. For this reason, the lack of EV-D68 in the CSF of AFM cases does not weaken plausibility. A mouse study showed that EV-D68 has tropism for spinal cord motor neurons and does not replicate efficiently in brain or other tissues [47]. The clinical syndrome in mice with EV-D68-induced paralysis shows a lower motor neuron pattern, with the upper limbs more affected and, corresponding to pathological findings, no cerebral or sensory involvement. This is consistent with AFM and supports EV-D68 causing AFM in humans.

The available evidence on EV-D68 and published knowledge about EVs in general, suggested that the criterion of plausibility was met.

### Coherence

The criterion for coherence was met because the current hypothesis that there is an association between EV-D68 and AFM did not contradict any prior knowledge about EV-D68. EVs have been shown to affect the nervous system and can cause other neurological complications [10,57,58]. Specifically, EV-A71 has been associated with severe neurological complications including AFM and encephalomyelitis during outbreaks of HFMD [48]. The available evidence suggested that this criterion was met.

### Experiment

There is some experimental evidence regarding the pathogenesis of EV-D68, but this is not specific to AFM. As mentioned in the section on biological gradient above, an experiment in mice showed that one strain produced weakness, limb tremors, paralysis and death, demonstrating neurovirulence of EV-D68 [41].

Recent studies showed genetic differences between EV-D68 strains from China, where outbreaks with milder disease occurred in 2015 and 2016, and the EV-D68 strains from the more severe US 2014 outbreak [59]. In addition, genetic analysis of EV-D68 strains from the US 2014 outbreak demonstrated that most strains belonged to clade B which is associated with severe disease and had not been detected in the US before the outbreak [30]. Analysis of EV-D68 strains globally indicated increasing genetic diversity and the evolution of clade B1 since 2000 [47].

A mouse study published in 2017 showed that strains of EV-D68 from the 2014 epidemic induced AFM in mice similar to the human clinical syndrome, with virus detected in the motor neurons [47]. The authors also showed that phylogenetically older strains (such as the Fermon strain) did not cause AFM. While only clade B1 has been isolated from human AFM patients [12], an experimental mouse study [47] identified neurovirulent strains from multiple clades (A, B, and B1). The study further demonstrated that Koch’s postulates were fulfilled when infecting naïve mice with strains from affected mice. Antibodies from infected mice protected naïve mice. The study also showed that viral RNA was detectable in tissue and CSF longer than infectious viral particles which do not grow well or persist for long in cerebral tissue and CSF. Intramuscular introduction of the virus resulted in 100% of the mice becoming paralysed, in contrast to much lower rates following intranasal or intraperitoneal inoculation.

The available evidence suggested that the experiment criterion was met.

### Analogy

This criterion was met in this investigation of causation, as there is suggestive evidence of AFM being associated with other EVs including EV-A71 and EV-D70 [10]. EV-A71 has been reported as one of the most important EVs capable of infecting nerve cells and has caused numerous outbreaks of paralytic disease [60,61]. In the Asia-Pacific region, a number of outbreaks of HFMD with associated complications such as AFM have been reported. A descriptive study was conducted following one such outbreak in Taiwan in 1998 in which more than 55 children died. Of 41 patients with neurological complications and EV-A71 infection, four had AFM. EV-A71 has also been shown to lead to complications such as aseptic meningitis, brain-stem encephalitis and rhombencephalitis [10].

The available evidence suggested that the criterion of analogy was met.

## Discussion

Historically, the incidence of EV-D68 has been low, with sporadic cases and small clusters of mild illness reported. Whether this represents under-ascertainment or true low incidence in the past is unclear, but active EV surveillance studies in several countries including Germany in 2013-14, Hong Kong in 2014, France in 2014 and China in 2011-15 suggest that EV-D68 was a rare cause of clinical infection in the past [18-21]. Since 2014, the number of reported infections and clusters has increased. In addition, severe complications including AFM have been reported since 2014 [3]. Several clusters of AFM in recent years were associated with EV-D68 and a large outbreak of EV-D68 in 2014 in the US was associated with severe respiratory illness.

Our application of the Bradford Hill criteria suggested good evidence for EV-D68 being a cause of AFM. While EVs in general are neurotropic, AFM has never previously been associated with EV-D68. It could be that incidence of EV-D68 was genuinely been much lower in the past, so that rare complications of infection have not been apparent. An analogous example is the association between Zika virus and microcephaly which was only recognised during a large-scale epidemic in Brazil in 2015 [62,63]. However, retrospective analysis of a large outbreak of Zika virus in French Polynesia two years earlier showed the same association with microcephaly but was not recognised at the time [64]. In addition, clades A, B and B1 of EV-D68 were highly neurovirulent in animal studies, with specific tropism for motor neurons [47]. It appears that these strains which evolved after the year 2000 are capable of causing AFM, as demonstrated in a mouse model, while the original Fermon and Rhyne strains do not cause AFM [47].

There is a need for phylogeographic epidemiology to ascertain temporal and geographic changes in the virus and whether such changes could explain why AFM is newly associated with the virus. Genetic changes in the virus which have rendered it more neuropathic could explain the association, and several clades have been shown to be highly neurovirulent. Phylogenetic studies have demonstrated that strains isolated in recent outbreaks are very divergent from the original Fermon strain isolated in 1962 [15]. Clades B1 and B2 caused the 2014 outbreak [30] and clade B3 caused an outbreak of severe EV-D68 infection in the Netherlands in 2016 [17]. Strains in clade B1 have mutations in structural and non-structural proteins, which could play a role in the reported neurovirulence of these strains [12], and all EV-D68-infections in human AFM cases were attributed to clade B. However, mouse studies showed that multiple clades (A, B and B1) cause paralysis [47]. The observation that clade B1 was associated with AFM in 2014 may be due to the much higher incidence of clade B1 infection in 2014. More research is needed to study biological gradient and to quantify measures of association between EV-D68 and AFM.

Given that the association of AFM with EV-D68 is recent, there is a strong case for systematic and enhanced EV surveillance, which will enable investigation of epidemiological data for measures of association. While past studies and EV surveillance showed that EV-D68 was a rare cause of EV infection, there has been a change in disease epidemiology since 2014, including a rise in the incidence of clade B infections. The lack of association between AFM cases and EV-D68 in the US in 2015 and 2016 [11] does not detract from our analysis, as AFM is a clinical syndrome with multiple possible aetiologies. More recent AFM cases could be due to a different aetiology, as other EVs continue to cause AFM, or could reflect the difficulty in isolating the virus from tissue and CSF. The Bradford Hill criteria are a tested and systematic method for evaluating causality and could be applied to other EVs.

## Conclusion

In summary, the application of the Bradford Hill criteria suggests that EV-D68 causes AFM. AFM has not previously been associated with EV-D68, and a mouse model shows that the original Fermon strain does not cause AFM, whereas the 2014 outbreak strain does [47]. It appears that the incidence of this infection and the clade-specific epidemiology have changed. Phylogeographic epidemiology will further our understanding of the temporal and spatial spread of increasingly neurovirulent clades and improve risk analysis. Further investigation into this relationship is important because of the severity of AFM, ongoing outbreaks of AFM and because there is currently no treatment for AFM related to EV-D68, and no vaccine to prevent infection [24,65].
